# Metal-dependent SpoIIE oligomerization stabilizes FtsZ during asymmetric division in *Bacillus subtilis*

**DOI:** 10.1371/journal.pone.0174713

**Published:** 2017-03-30

**Authors:** Ewa Cendrowicz, Anabela de Sousa Borges, Malgorzata Kopacz, Dirk-Jan Scheffers

**Affiliations:** Department of Molecular Microbiology, Groningen Biomolecular Sciences and Biotechnology Institute, University of Groningen, Groningen, the Netherlands; Centre National de la Recherche Scientifique, Aix-Marseille Université, FRANCE

## Abstract

SpoIIE is a bifunctional protein involved in asymmetric septum formation and in activation of the forespore compartment-specific transcription factor σ^F^ through dephosphorylation of SpoIIAA-P. The phosphatase activity of SpoIIE requires Mn^2+^ as a metal cofactor. Here, we show that the presence of a metal cofactor also influences SpoIIE oligomerization and asymmetric septum formation. Absence of Mn^2+^ from sporulation medium results in a delay of the formation of polar FtsZ-rings, similar to a *spoIIE* null mutant. We purified the entire cytoplasmic part of the SpoIIE protein, and show that the protein copurifies with bound metals. Metal binding both stimulates SpoIIE oligomerization, and results in the formation of larger oligomeric structures. The presence of SpoIIE oligomers reduces FtsZ GTP hydrolysis activity and stabilizes FtsZ polymers in a light scattering assay. Combined, these results indicate that metal binding is not just required for SpoIIE phosphatase activity but also is important for SpoIIE's role in asymmetric septum formation.

## Introduction

In response to starvation conditions, *Bacillus subtilis* cells cease vegetative growth and initiate the formation of a dormant cell type called a spore [1–3]. The first visible step in this process is the formation of the asymmetrically positioned septum that divides the cell into two daughter cells of different size: the larger mother cell and the smaller forespore, that each receive one copy of the chromosome. Subsequently, compartment specific transcription factors σ^F^ and σ^E^ are activated in the forespore and in the mother cell, respectively. This activation event is critical because it initiates the rest of the sporulation developmental program in each daughter cell. Ultimately, the forespore becomes the spore and the mother cell lyses when the process is complete.

**Fig 1:**
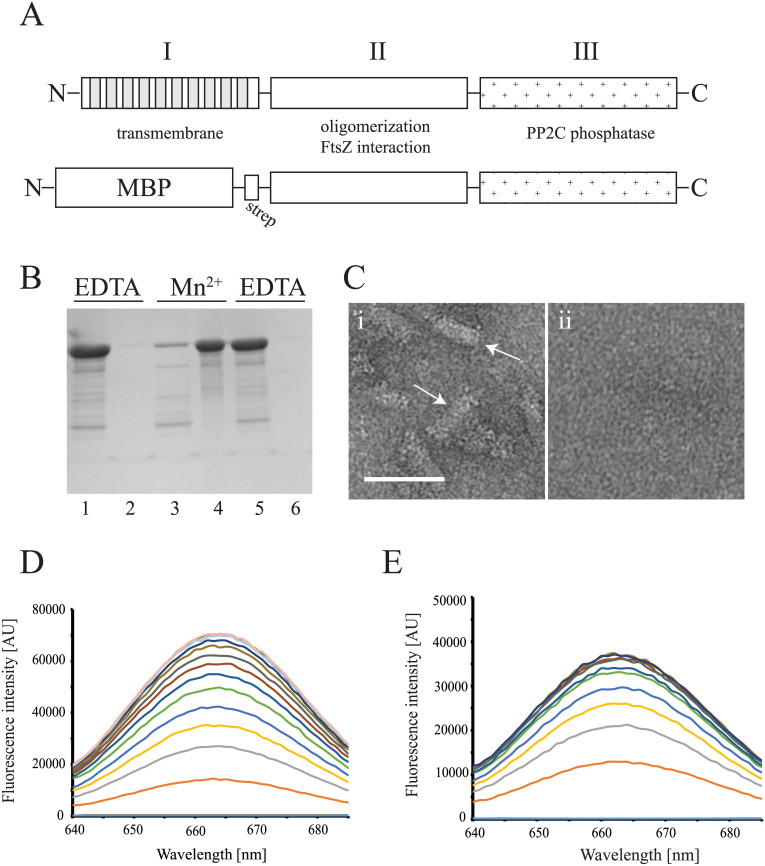
(A) The putative three domain structure of SpoIIE (top) and a schematic of the SpoIIE construct used in this study (bottom), (B) SDS-PAGE of the sedimentation of ms-SpoIIE_cyt_. Ms-SpoIIE_cyt_ was pre-incubated with 1 mM EDTA (lanes 1 and 2), followed by the incubation with 5 mM MnCl_2_ (lanes 3 and 4), followed by the incubation with 10 mM EDTA (lanes 5 and 6). Odd numbers below the image represent supernatant fractions and even numbers represent pellet fractions, (C) Electron microscopy of ms-SpoIIE_cyt_ oligomers after co-purification with Fe^2+^ (i). Oligomers were not observed after incubation of the protein with 5 mM EDTA (ii). Arrows point to rod and circular structures of ms-SpoIIE_cyt_. Scale bar: 50 nm, (D, E) Fluorescence emission spectra of s-SpoIIE_cyt_-Cy5 titrated into a buffer in the absence (D) and presence (E) of Mn^2+^. Every spectrum represents a titration step with a 0.23 μM concentration increase of s-SpoIIE_cyt_-Cy5, with the bottom spectrum representing buffer without any added protein.

Upon entry into sporulation, the cell division protein FtsZ, that during vegetative growth drives the mid-cell division, switches its position to the polar sites of the cell. The switch is triggered by an integral membrane protein called SpoIIE [4–7]. At the onset of sporulation, SpoIIE co-localizes with FtsZ at mid-cell and both proteins redeploy to polar sites via a spiral-like intermediate [4]. Recently, it was shown that this process also requires DivIVA, which interacts with SpoIIE [8]. Next, one of the polar Z-rings disassembles and the other one is converted into a division septum. SpoIIE contributes to, but is not essential for, the formation of polar Z-rings. In *spoIIE* null mutant cells, FtsZ still localizes to the polar sites but the switch from medial to polar rings is delayed and the frequency of polar Z-ring formation is decreased [9, 10]. While the FtsZ ring constricts, DivIVA and SpoIIE remain localized at the 'base' of the septum, with SpoIIE predominantly located at the prespore side of the septum [8]. SpoIIE is then transferred from the septum to the cell pole of the prespore, primed to perform its second function, while SpoIIE in the mothercell is degraded by FtsH [11]. This second SpoIIE function is the activation of the prespore specific transcription factor σ^F^ by dephosphorylating the anti-sigma factor antagonist SpoIIAA [12–14]. SpoIIE phosphatase is inactive in the pre-divisional cell and becomes active only after the asymmetric septum is formed [15]. It is thought that FtsZ may be involved in the activation of SpoIIE phosphatase, as well as oligomerization of SpoIIE, which protects SpoIIE from proteolysis by FtsH [11]. SpoIIE is a transmembrane protein with a three-domain structure: the N-terminal domain (domain I) consists of 10 membrane-spanning segments; the poorly conserved central domain (domain II) is involved in the oligomerization of SpoIIE and its interaction with FtsZ; the C-terminal domain (domain III) is the phosphatase domain (Fig 1A) [16–19]. There is a clear structural separation of the three domains of SpoIIE. The C-terminal domain III can independently function as a phosphatase *in vitro* [20], whereas the central domain II can interact with either FtsZ or itself [21], independent of the other two domains. However, some mutations in domain II influence the phosphatase activity of SpoIIE [15], whereas mutations in domain III that impair the phosphatase activity also impair polar cell division [7]. The structure of domain III revealed that this domain is structurally related to the Protein phosphatase 2C (PP2C), Mn^2+^-dependent family of protein phosphatases. Crystallized domain III contained one bound Mn^2+^ ion at low occupancy even though crystals were grown in the presence of 50 mM MnCl_2_, suggesting very weak Mn^2+^ binding [19]. This was confirmed by the inability to detect bound Mn^2+^ by atomic absorption spectroscopy or size exclusion chromatography [19]. The phosphatase activity of domain III is Mn^2+^ dependent but could only be detected in the presence of high concentrations (20 mM) of added MnCl_2_, again indicating that Mn^2+^ is not bound with high affinity [19]. Interestingly, other PPP2C family members contain two or three Mn^2+^ ions, and SpoIIE has the equivalent residues to coordinate a second Mn^2+^ ion although this second ion was not found in the structure [19].

SpoIIE and FtsZ have been shown to interact *in vitro* using gel filtration [21]. However, details about the SpoIIE-FtsZ interaction and SpoIIE self-interaction are not known due to difficulties in the expression and purification of the soluble cytoplasmic domain of SpoIIE [20].

Here, we purified the soluble part of SpoIIE (SpoIIE_cyt_) as a fusion protein and show that SpoIIE oligomerization is greatly stimulated by the presence of metal ions, which in turn stabilizes FtsZ polymers. Further, we show that in the absence of manganese, the formation of asymmetric septa is delayed, similar to the reported *spoIIE* null phenotype. Our data indicate that metal binding is not just required for the phosphatase activity of SpoIIE, but also for its interaction with FtsZ.

## Methods

### Plasmids and strains

pMalC2x (New England Biolabs) was used to clone and purify strep-tagged-SpoIIE_cyt_ (s-SpoIIE_cyt_) as an N-terminal MBP-strep-tag-SpoIIE_cyt_ (ms-SpoIIE_cyt_) fusion protein (Fig 1A). A 1503 bps fragment coding for *spoIIE*_*cyt*_ was amplified by PCR from *B*. *subtilis* 168 template genomic DNA using a forward primer containing the strep-tag coding sequence (bold) (5'-**AGCGCTTGGCGTCACCCGCAGTTCGGTGGT**CCTCAATCTATTACGAGGAAAGTGG)[15] and a reverse primer containing a *Bam*HI site (underlined) (5'-GCGGATCCCATATATTCCCATCTTCGCCAGAAG). The PCR product was digested using *Bam*HI and ligated into pMalC2x linearized with *Xmn*I (blunt end)/*Bam*HI, resulting in plasmid pEK33. The construct was verified by sequencing.

*B*. *subtilis* strains are listed in Table 1. Strain 4120 was constructed using standard *B*. *subtilis* transformation methods [22].

**Table 1:** *B*. *subtilis* strains used in this study.

strain	relevant genotype	reference/construction
168	wild type, *trpC2*	lab collection
4055	*trpC2 amyE*::*spc P*_*hyperspac*_*-ftsZ-eyfp*	[23]
4120	*trpC2 amyE*::*spc P*_*hyperspac*_*-ftsZ-eyfp lacA*::*P*_*xyl*_*-kinA erm*	IDJ015 transformed to spectinomycin resistance with 4055 chromosomal DNA.
IDJ006	*P*_*spo0A*_*-gfp*, *Cm*^*R*^	[24]
IDJ007	*P*_*spoIIA*_*-gfp*, *Cm*^*R*^	[24]
IDJ015	*trpC2 lacA*::*Pxyl-kinA erm*	[24]

### Protein expression and purification

FtsZ was purified as described, to > 95% purity [25]. Ms-SpoIIE_cyt_ or MBP alone were produced in *E*. *coli* BL21-RIL cells. Freshly transformed cells were grown overnight on LB (Lysogeny Broth, Lennox) agar plates containing 100 μg/ml ampicillin and 50 μg/ml chloramphenicol. Liquid LB medium containing the same antibiotics was inoculated with a single colony from a fresh plate. An overnight culture was diluted 1:100 into fresh LB containing the same antibiotics and grown at 37°C until OD_600_ = 0.7, when iso-propyl-β-D-thiogalactopyranoside (IPTG) was added to 1 mM to produce the fusion proteins, and growth was continued at 37°C for 3 hours, after which cells were washed once with Tris-buffered Saline (TBS, 50 mM Tris/HCl, pH 7.6; 150 mM NaCl) and harvested by centrifugation. Cell pellets were flash frozen and stored at -20°C. For purification, cells were resuspended in buffer A (50 mM Tris/HCl pH = 7.5, 300 mM KCl, 0.5 mM DTT) supplemented with 0.1% Triton X-100 and disrupted by sonication. The cell lysate was clarified by centrifugation at 20,000 x *g* for 20 min and the supernatant was applied onto amylose resin (5 ml column volume) previously equilibrated with buffer. The resin was washed with the same buffer without Triton X-100 and the protein was eluted with 50 mM Tris/ HCl pH = 7.5, 300 mM KCl, 0.5 mM DTT and 10 mM maltose. All proteins were concentrated using Amicon Ultra-15 Centrifugal Filter Units (Merck Millipore). Protein concentrations were measured and absorption spectra were taken using a NanoDrop ND-1000 Spectrophotometer (ISOGEN Lifescience), and the protein was stored at -80°C. The extinction coefficient for ms-SpoIIE_cyt_ (Ex = 107720 M^-1^cm^-1^) was calculated using the ExPASy ProtParam tool [26]. Ms-SpoIIE_cyt_ was purified to > 95% purity based on SDS-PAGE analysis (not shown), however the protein was quite labile resulting in some degradation during incubations (e.g. Fig 1B).

To obtain s-SpoIIE_cyt_, the protein solution was mixed with an equal volume of 50 mM Hepes/ NaOH pH = 7.5, 1M KCl, 0.5mM DTT, 1mM EDTA and 1% Triton X-100 to obtain final concentrations of 650 mM KCl and 0.5% Triton X-100. The MBP tag was cleaved overnight at 4°C using Factor Xa protease (New England Biolabs). Factor Xa was deactivated using Dansyl-glu-gly-arg-chloromethyl ketone and the protein mixture was applied onto HiLoad Superdex 16/600 gel filtration column and eluted with 50 mM Hepes/ NaOH, pH = 7.5, 650 mM KCl, 0.5 mM DTT, 1 mM EDTA and 0.5% Triton X-100.

### ICP-OES measurement

Purified ms-SpoIIE_cyt_ was lyophilized and analysed for the presence of calcium, iron, magnesium, manganese and zinc using inductively-coupled plasma optical emission spectroscopy on an Optima 7000DV ICP-OES (PerkinElmer) apparatus. The measurements were performed in duplicate using protein obtained during two independent purifications.

### Light scattering

The effects of various cations on the oligomerization of s-SpoIIE_cyt_ were monitored by 90° light scattering using an AMINCO-Bowman Series 2 fluorescence spectrometer. s-SpoIIE_cyt_ or MBP (1.5 μM) were incubated in 50 mM MES/NaOH pH = 6.5, 300 mM KCl. After 60 seconds of incubation various chloride salts of divalent cations (Ca^2+^, Mg^2+^, Mn^2+^, Zn^2+^, Fe^2+^, Cu^2+^, Co^2+^, Ni^2+^) were added to the solution (10 mM final concentration) and the light scattering signal was monitored for 1 hour.

EDTA mediated reversal of s-SpoIIE_cyt_ oligomerization was performed in a larger volume cuvette (1 ml) to allow stirring, and measurements were taken using a QuantaMaster^™^ spectrofluorometer controlled by the FelixGX program (Photon Technology International, Inc.). MnCl_2_ (10 mM final concentration) was added to the sample after 3.5 min and EDTA (20 mM final concentration) was added after 10 minutes of incubation. As a dilution control, an equal volume of H_2_O was added to a duplicate sample. The light scattering signal of protein without Mn^2+^ was measured as blank.

The effect of s-SpoIIE_cyt_ (1.5 μM) on the assembly of FtsZ (10 μM) was studied in 50 mM Tris/HCl, pH = 7.5, 300 mM KCl, 10 mM MgCl_2_. After 90 sec of measurement GTP or GDP (2 mM final concentration) was added to the sample. Samples without s-SpoIIE_cyt_ contained an equal volume of storage buffer. As controls, measurements were taken of strep-SpoIIE_cyt_ and/or FtsZ in the presence or absence of nucleotides. All experiments were done at 30°C.

### Oligomerization of fluorescent ms-SpoIIE_cyt_

To study the oligomerization of ms-SpoIIE_cyt_ in the presence and absence of Mn^2+^, the fluorescence spectra of ms-SpoIIEcyt-Cy5 were acquired in the presence of Mn^2+^ or EDTA. ms-SpoIIE_cyt_ was purified from lysates by binding to an amylose resin as described above, with the inclusion of an on-column Cy5 labeling step. After washing with buffer A, the resin with bound ms-SpoIIE_cyt_ was mixed with fluorescent label Cy5 (GE Healthcare) and incubated for 1 hour at 4°C. Excess label was washed away using the same buffer and labeled protein was eluted with buffer A supplemented with 10 mM maltose.

Equal volumes of ms-SpoIIE_cyt_-Cy5 were titrated to a buffer containing 50 mM Tris/HCl, 300 mM KCl, 1 mM EDTA with or without 10 mM MnCl_2_, at 30°C. Each titration step increased the total protein concentration with 0.23 μM. Immediately after each addition of ms-SpoIIE_cyt_, a fluorescence spectrum (640–685 nm, excitation 633 nm) was recorded in a QuantaMaster^™^ spectrofluorometer controlled by the FelixGX program (Photon Technology International, Inc.). Oligomerization was monitored by Cy5 self-quenching and the titration was stopped when fluorescence no longer increased upon the addition of protein.

### Sedimentation assay

To study metal dependent sedimentation of ms-SpoIIE_cyt_, the protein (10 μM) was incubated in a total volume of 100 μl of buffer (50 mM MES/ NaOH, pH = 6.5, 50 mM KCl) supplemented with 1 mM EDTA. After 20 min, a 30 μl aliquot was collected and the remaining 70 μl was supplemented with 5 mM MnCl_2_ and incubated for another 30 min. Another 30 μl aliquot was collected and the rest of the sample was supplemented with 10 mM EDTA and incubated for another 40 min before taking the final aliquot. All aliquots were spun down, immediately after collection, at 186,000 x *g* and the supernatants and pellets were separated for analysis by SDS-PAGE.

### Electron microscopy

To visualize ms-SpoIIE_cyt_ using Transmission Electron Microscopy (TEM), 10 μM ms-SpoIIE_cyt_ was prepared in 50 mM Hepes/ NaOH, pH = 7.5, 50 mM KCl.

To visualize FtsZ in the presence or absence of s-SpoIIE_cyt_, 10 μM FtsZ with or without 1.5 μM s-SpoIIE_cyt_ was prepared in polymerization buffer: 50 mM Tris/ HCl, pH = 7.5, 300 mM KCl supplemented with 1 mM EDTA or 10 mM MgCl_2_ or 10 mM MnCl_2_. After 5 min of incubation at 30°C, 1 mM GTP or 1 mM GDP was added to the mixture. Additionally, 1.5 μM s-SpoIIE_cyt_ was prepared as above in the presence of 10 mM MnCl_2_. 2 μl of each sample was collected after 30 min and applied onto glow discharged carbon grids prepared as described [25]. The grids were examined in a Philips CM120 electron microscope equipped with a LaB_6_ filament operating at 120 kV. Images were recorded with a Gatan 4000 SP 4 K slow-scan CCD camera at magnifications 35,000x (for FtsZ ± s-SpoIIE_cyt_) or 45,500x (for ms-SpoIIE_cyt_ structures alone).

### Fluorescence microscopy

Overnight culture of *B*. *subtilis* strain 4055 (*P*_*hyperspac*_ *-ftsZ-eyfp*) [23] or 4120 (*P*_*hyperspac*_ *-ftsZ-eyfp*, *P*_*xyl*_*-kinA*) grown in CH medium [27] was diluted into fresh CH to an OD of 0.1. Cells were grown at 37°C until OD of 0.7. At this point, 2 samples of 5 ml were taken and cells were collected and washed 2 times with the same volume of CH with (spo+) or without (spo-) manganese. After the washing steps, spo+ and spo- cells were resuspended in 100 μl of CH with and without manganese, respectively. Sporulation medium [28](with or without manganese) was added up to the volume of 5 ml in the presence of 0.02 mM of IPTG to allow low level expression of *ftsZ-eyfp*. In the case of strain 4120, 0.5% (w/v) Xylose was also added to the sporulation medium to overproduce KinA. Cells were allowed to sporulate at 37°C by continuing the incubation. Every hour, a 500 μl sample was taken and cells were harvested and resuspended in 20–50 μl of PBS before being mounted on an agarose pad prior to microscopy. FtsZ-eYFP was visualized as described in [23] and cells were scored according to their Z-ring localization.

### Promoter-reporter fusion monitoring

Strain 168 (control), IDJ006 (*P*_*spo0A*_*-gfp*) and IDJ007 (*P*_*spoIIA*_*-gfp*) were grown and induced for sporulation exactly as described above. Upon resuspension in sporulation medium with or without manganese, the cells were transferred to a 96 well plate, 150 μL of culture per well. The plate was incubated in a Biotek Synergy Mx Microplate reader and cells were allowed to sporulate by incubation at 37°C with continuous shaking. Growth and GFP production were monitored every 10 min for 8 hours by measuring absorbance (at 600 nm) and fluorescence (excitation 485 nm; emission 528 nm.; both 20 nm bandpass). Promoter activity was calculated as the ratio of fluorescence over absorbance (to correct for cell density). Two biological replicates were tested, each in triplicate.

### GTPase assay

Real-time coupled GTP hydrolysis assays were performed as described in [29, 30]. The reaction mixture contained 20 U/ml pyruvate kinase/lactate dehydrogenase mixture (Sigma Aldrich), 2 mM phospho(enol)pyruvate (PEP, Sigma Aldrich), 1 mM NADH (Sigma Aldrich), 10 μM FtsZ and no or 1.5 μM s-SpoIIE_cyt_ in polymerization buffer: 50 mM Tris/HCl, pH = 7.5, 300 mM KCl. The reaction was started after 2 min of incubation at 30°C with 1 mM GTP and 10 mM MgCl_2_ mixture in the same polymerization buffer, in order to avoid oligomerization of s-SpoIIE_cyt_ before polymerization of FtsZ. The reaction was followed for 60 min at 30°C.

## Results

### Metal binding enhances SpoIIE oligomerization

A 501 residue fragment of SpoIIE containing domains II and III (amino acids 326–827) was N-terminally fused to maltose binding protein (MBP) via a short strep-tag (Fig 1A). The ms-SpoIIE_cyt_ protein was purified with a high yield (~ 40 mg/1L culture). The absorption spectrum of purified ms-SpoIIE_cyt_ showed a broad peak in the 420 nm region (S1A Fig), similar to those of iron/manganese-protein complexes [31], suggesting that ms-SpoIIE_cyt_ was purified in a metal bound form. Domain III of SpoIIE belongs to the Mn^2+^- dependent phosphatase 2C family of phosphatases and is capable of binding one Mn^2+^ ion when the protein is crystallized in the presence of excess MnCl_2_ [19]. Ms-SpoIIE_cyt_ was lyophilized and bound metals were analyzed by inductively-coupled plasma optical emission spectroscopy, which revealed that ms-SpoIIE_cyt_ copurifies with iron bound in a metal/protein ratio close to 2:1 (Table 2). The analysis also revealed small amounts of calcium and magnesium bound to the protein but manganese levels were below the detection limit (Table 2), even when the growth medium was supplemented with 0.5 mM MnCl_2_ (not shown). This is in agreement with a previous study in which bound Mn^2+^ could not be detected by atomic absorption spectroscopy or size exclusion chromatography after purification of domain III [19].

**Table 2:** Metals copurifying with ms-SpoIIE_cyt_. Metals associated with lyophilized ms-SpoIIE_cyt_ were analyzed by ICP-OES. The mean and standard deviation from two independent determinations are shown.

metal	Fe	Ca	Mg	Mn	Zn
Average ratio of metal to protein	2,033 ± 0,119	0,450 ± 0,099	0,030 ± 0,020	0	0,008 ± 0,013

It was possible to replace iron with manganese by incubation of the protein with EDTA followed by incubation with excess of MnCl_2_. After incubation with EDTA, the absorption peak at 420 nm disappeared (S1B Fig). The absorption peak re-appeared when the protein was incubated with excess MnCl_2_ (S1C Fig). Domain II (amino acids 321–567, Fig 1A) is involved in the oligomerization of SpoIIE [21]. To determine whether metal binding influences SpoIIE oligomerization, we repeated the metal exchange experiment with EDTA and MnCl_2_ and used high speed centrifugation to separate higher order oligomers from soluble protein. After incubation with EDTA, ms-SpoIIE_cyt_ was found in the supernatant (Fig 1B). Subsequent incubation with MnCl_2_ resulted in sedimentation of a large fraction of the protein, which upon resuspension in the presence of EDTA returned to a soluble form (Fig 1B). This shows that ms-SpoIIE_cyt_ is capable of metal-dependent oligomerization. Oligomerization is reversible upon EDTA incubation indicating that the sedimentation is not the result of aspecific protein aggregation. Purified protein, when examined by EM, also showed larger structures suggestive of protein oligomers, which were not detected after incubation of the protein with EDTA (Fig 1C).

SpoIIE is also able to form small oligomers (up to 300 kDa as determined by gel filtration chromatography) in the absence of added metal ions [21], however these oligomers cannot be observed in sedimentation and light scattering (below) assays. To test whether the presence of metals influences the formation of small oligomers of SpoIIE, ms-SpoIIE_cyt_ was labeled with the fluorescent dye Cy5. Cy5 self-quenching has been used to detect oligomerization/clustering of proteins, such as v-SNAREs [32]. Cy5-labeled ms-SpoIIE_cyt_ was titrated into a buffer supplemented with either EDTA or MnCl_2_, and the increase in fluorescence signal was followed until the signal ceased to increase (Fig 1D and 1E). The quantum yield of ms-SpoIIE_cyt_-Cy5 was lower in the presence of metal compared to the samples with EDTA (note difference in scale between 1D, E), possibly due to binding of the protein to metal ions or formation of dimers. However, the goal was not to compare the absolute intensities of the spectra between the two experiments but to find the concentration at which fluorescence did not increase as a result of self-quenching which we interpret to be due to the formation of small oligomers of ms-SpoIIE_cyt_-Cy5. The stop in signal increase occured at a lower protein concentration (1.4 μM) when ms-SpoIIE_cyt_-Cy5 was incubated with Mn^2+^ than when ms-SpoIIE_cyt_-Cy5 was incubated with EDTA (2.1 μM). Although these experiments do not reveal the size of the oligomers, nor the absolute critical concentration for oligomerization, these results do suggest that the concentration at which small ms-SpoIIE_cyt_ oligomers start to form is lowered in the presence of metals.

**Fig 2:**
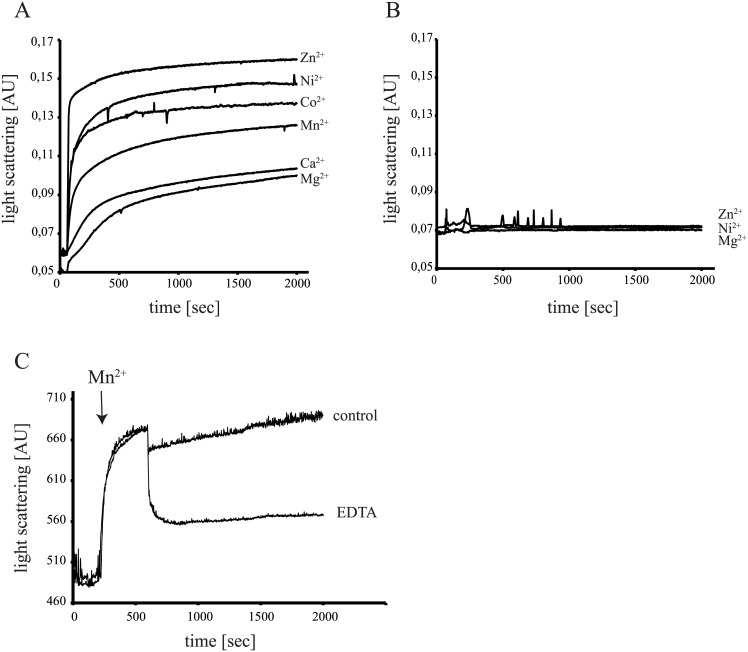
Binding to divalent cations reversibly enhances oligomerization of s-SpoIIE_cyt_. (A, B) Light scattering measurement of 1,5 μM s-SpoIIE_cyt_ (A) and MBP (B) in the presence of 10 mM of divalent cations added after 60 sec. (C) Light scattering signal of s-SpoIIE_cyt_, with 10 mM Mn^2+^ added after 60 sec and 20 mM EDTA or H_2_O (control) added after 600 sec.

To study the influence of metal ions on SpoIIE_cyt_ oligomerization in more detail, we removed the MBP-tag from the fusion protein via proteolytic cleavage resulting in s-SpoIIE_cyt_ which only contains a short 10 amino acid N-terminal strep-tag. Oligomerization of s-SpoIIE_cyt_ in the presence of its cofactor Mn^2+^ and other divalent cations was followed by light scattering. All divalent cations induced the oligomerization of s-SpoIIE_cyt_ (Fig 2A), whereas buffer had no effect (S2 Fig). Fe^2+^ could not be tested as Fe^2+^ is oxidized very easily in solution which made the analysis in the presence of Fe^2+^ impossible even when reducing agents like DTT or ascorbate were added (not shown). The maximum light scattering signal obtained in the presence of different metals reflected the preferential binding of metal ions by proteins described by the Irving-Williams series (Mn(II) < Fe(II) < Co(II) < Ni(II) < Cu(II) > Zn(II) [33]), and is in line with the observation that the metal-binding preferences of metalloproteins most often do not match the metal requirement of the protein [34]. Thus, it is tempting to speculate that the observed oligomerization is the result of the affinity of SpoIIE for the metals tested. An alternative explanation that would fit these results is that the different metals affect the oligomerization of SpoIIE differently. In a control experiment, incubation of MBP with metals did not result in an increase in light scattering (Fig 2B). As in the sedimentation experiment, the metal induced oligomerization of s-SpoIIE_cyt_ could be (largely) reversed by the addition of EDTA (Fig 2C).

### The absence of manganese delays polar Z-ring formation *in vivo*

**Fig 3:**
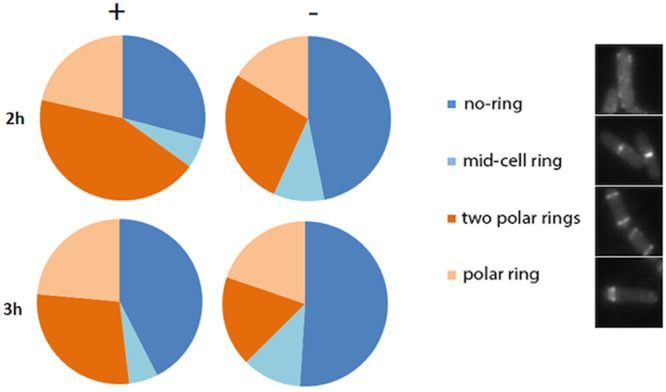
The absence of Mn^2+^ from the sporulation medium delays asymmetric Z-ring formation. Pie-chart representation of sporulating *B*. *subtilis* cells in the presence (+) and absence (-) of Mn^2+^. Four different types of cell (representative images in the legend) were scored: cells without any Z-ring in the cell (no ring), cells with a Z-ring in the middle of the rod (mid-cell ring), cells with two rings assembled at the cell poles (two polar rings) and cells with only one polar ring (polar ring). Non-sporulating cells are marked in blue shades, while sporulating cells are marked in orange shades. Each pie chart is the result of two independent classification experiments in which at least 290 cells were classified per condition. Actual percentages and standard deviations are included in S1 Table.

The dependence of the sporulation process on manganese is well known, but has so far not been linked to efficient formation of the sporulation septum. The best known role of manganese is its requirement as a cofactor in the phosphatase activity of SpoIIE [13]. Manganese could also influence the sporulation phosphorelay as Mn^2+^ has been reported to upregulate Spo0A phosphorylation through KinD [35], and manganese could function as a metal cofactor of Spo0F which is a crucial component of the phosphorelay [36, 37]. To test whether the role of SpoIIE in polar septation, which precedes its role as a phosphatase, is also metal dependent, we studied FtsZ-eYFP localization in sporulating *B*. *subtilis* cells in the presence and absence of manganese. In the absence of manganese, relocation of the Z-ring from mid-cell to the cell poles is delayed (compared to samples supplemented with Mn^2+^). We noticed that in the absence of Mn^2+^ cells contain more mid-cell rings and significantly less polar rings compared to cells in sporulation medium supplemented with Mn^2+^ (Fig 3). It is clear that asymmetric septum formation is affected in the absence of manganese. To test whether the sporulation phosphorelay is affected in the absence of manganese, we investigated the activity of the *spo0A* and *spoIIA* promoters in cells sporulating in the presence and absence of manganese (S3 Fig). This showed that P_spo0A_ activity is not changed, which is interesting as Spo0A drives the relocalization of FtsZ to the poles [38]. On the other hand, P_spoIIA_ activity, the outcome of the phosphorelay, is delayed in the absence of manganese (S3 Fig).

To exclude, as much as possible, the possibility that the delay in the phosphorelay is the principal cause for the delay in polar septation in the absence of Mn^2+^, we repeated this experiment in a strain that overproduces KinA. KinA overproduction artificially charges the phosphorelay both in sporulating and even in exponentially growing cells [24]. To prevent overcharging the phosphorelay during growth, *kinA* was only induced after resuspension in sporulation medium. KinA overproduction resulted in faster sporulation, and therefore cells were analysed after 60, 120 and 180 mins. As above, the absence of Mn^2+^ resulted in more cells with mid-cell rings and less cells with polar rings after 60 mins (S4 Fig, S2 Table). After 120 mins, midcell rings had completely disappeared and the number of cells with polar rings was significantly lower in the absence of Mn^2+^ (S4 Fig, S2 Table). The total amount of cells with polar rings was also decreased compared to the initial experiment as polar FtsZ rings disassemble once the polar septum is formed, which occurs very fast under these conditions.

The delay and decrease in the formation of asymmetric Z-rings in the absence of manganese is similar to what is observed in *spoIIE* null mutants [9, 10]. Although this delay could be a secondary effect caused by a delay in the phosphorelay, polar septation is also reduced in the absence of manganese when the phosphorelay is artificially charged (S4 Fig). Combined, these results suggest that the role of SpoIIE in relocating FtsZ to the cell pole is affected in the absence of manganese.

### FtsZ polymers are stabilized by SpoIIE

**Fig 4:**
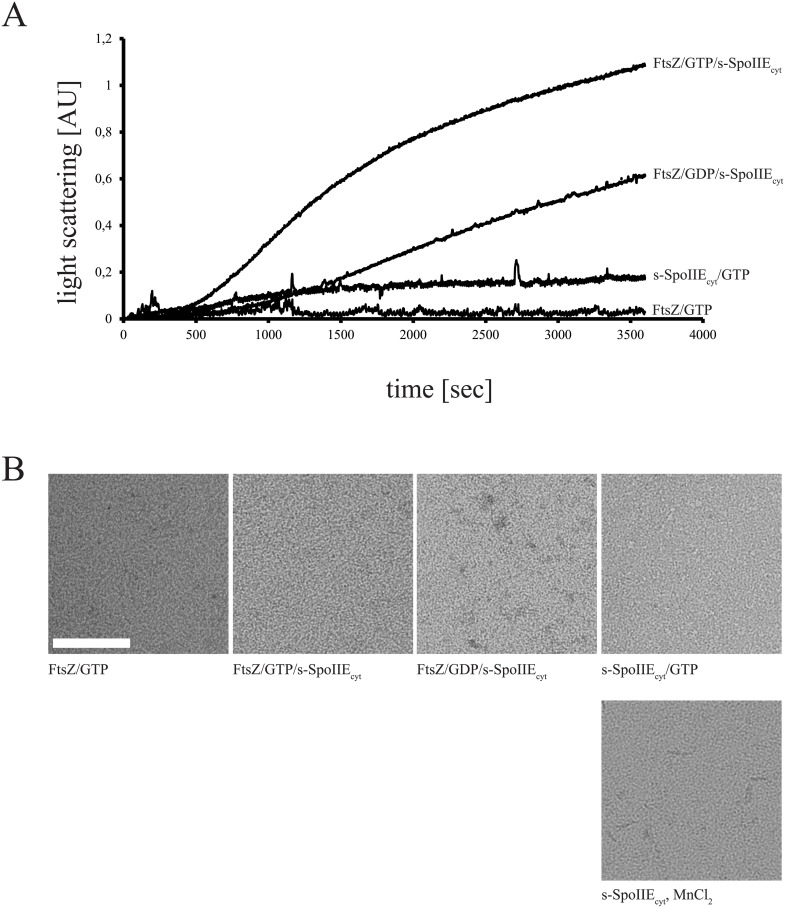
s-SpoIIE_cyt_ interacts directly with FtsZ. A) Light scattering signals of FtsZ (FtsZ/GTP) s-SpoIIE_cyt_ (s-SpoIIEcyt/GTP) or FtsZ and s-SpoIIEcyt (FtsZ/GTP/s-SpoIIEcyt) in the presence of GTP, or FtsZ and s-SpoIIEcyt in the presence of GDP(FtsZ/GDP/s-SpoIIEcyt). B) Electron micrographs of a similar experiment as shown in A), samples were taken after 30 min incubation and placed on EM grids. Scale bar, 100 nm.

FtsZ and SpoIIE are known to interact [21]. To study the effect of SpoIIE on FtsZ in more detail we analysed the dynamics of FtsZ polymerization in the presence of s-SpoIIE_cyt_. In control experiments, the presence of Mn^2+^ in the polymerization buffer resulted in FtsZ polymerization followed by irreversible bundling (S5A, S5C and S5F Fig) which caused FtsZ to come out of solution. As s-SpoIIE_cyt_ also oligomerizes with Mg^2+^ and this metal allows dynamic polymerization and depolymerization of FtsZ we chose to only include Mg^2+^ in the polymerization buffer. As expected, polymerization of FtsZ alone was dynamic and GTP dependent, although the signal is barely visible in the settings used in this experiment (Fig 4A, trace FtsZ/GTP, see also S5A Fig). S-SpoIIE_cyt_ alone in the polymerization buffer with GTP resulted in oligomerization as seen above (Fig 4A, trace s-SpoIIE_cyt_/GTP). However, when FtsZ and s-SpoIIE_cyt_ were both present, a strong signal increase could be observed which was more than the individual signals combined, suggesting that both proteins co-polymerize (Fig 4A). The increase in signal was independent of the nucleotide added although GTP caused the increase to occur after a shorter lag phase and to a higher signal over the 1 hr timecourse of the experiment. This result suggests that s-SpoIIE_cyt_ and FtsZ interact to form very stable polymers, perhaps bundles. Samples were taken after 30 min and examined by EM. Samples containing FtsZ alone did not show polymers as these polymers would have disassembled at this 30 min timepoint (Fig 4B, see also S5A and S5E Fig)—although our FtsZ was clearly capable of forming polymeric structures that were stable over 30 min when formed in the presence of EDTA, which blocks GTP hydrolysis and thus polymer disassembly (see S5D Fig). To our surprise, the samples containing s-SpoIIE_cyt_ and FtsZ did not contain bundles and even individual polymers were difficult to detect (Fig 4B), although the FtsZ concentrations used in this experiment were similar to previous experiments that easily identified FtsZ bundling by ZapA [39]. The structures observed in samples containing s-SpoIIE_cyt_ and Mn^2+^ were not as large as the ones observed with ms-SpoIIE_cyt_ and Fe^2+^ (Fig 1C), however this experiment is performed at a lower protein concentration, in a different buffer system, and the protein s-SpoIIE_cyt_ itself is smaller as it lacks the MBP-fusion tag (370 aa). Thus, these structures cannot be directly compared.

Stabilization of FtsZ polymers is concomitant with a reduction in FtsZ GTPase activity, as previously shown for ZapA bundling [40]. When FtsZ GTPase was monitored in the presence of s-SpoIIE_cyt_ under similar conditions, a strong reduction in GTPase activity was observed, from 2.22 ± 0.22 GTP/min/FtsZ without s-SpoIIE_cyt_ to 0.16 ± 0.03 GTP/min/FtsZ in the presence of s-SpoIIE_cyt_. This is in agreement with stabilization of FtsZ polymers by s-SpoIIE_cyt_.

## Discussion

SpoIIE is a bifunctional protein involved in asymmetric septum formation and activation of the forespore compartment-specific transcription factor σ^F^ [5, 13]. In this short paper we investigate the role of a metal cofactor in both functions of SpoIIE. We purified the entire cytoplasmic part of the SpoIIE protein, which consists of two domains that are involved in oligomerization and interaction with FtsZ (domain II) [21] and in dephosphorylation of SpoIIAA-P (domain III) [19]. Isolated domain III is sufficient to dephosphorylate SpoIIAA in the presence of the cofactor Mn^2+^ [19]. In the crystal structure of the phosphatase domain of SpoIIE, only a single manganese ion was found at low occupancy in the active site. This ion could only be detected in the structure after growing crystals in excess MnCl_2_, but not by other methods [19]. The presence of only one Mn^2+^ ion in the structure represented a fundamental difference between SpoIIE and the other type PP2C phosphatases, *eg*. human PP2Cα phosphatase which has two metal ions bound at the active site [19]. Our analysis revealed that ms-SpoIIE_cyt_ copurifies with two bound metal ions and that metal binding is involved in the reversible oligomerization of SpoIIE_cyt_. The presence of two metal ions is in agreement with the predictions for PP2C phosphatases and the presence of a second metal binding pocket predicted from the primary structure of SpoIIE [19]. There are various explanations for the difference in the amount of metal ions found in this study compared to the previous work [19]. Domain III was crystallized in Na-citrate which, due to its chelating properties, may have removed a less-tight bound metal from domain III. It is also possible that domain II of SpoIIE is necessary for the stable binding of a second metal by domain III, or that domain II itself is involved in the binding of the second metal (although the latter is not very likely). After removal of bound iron, metal dependent oligomerization followed the metal binding preference described in the Irving-Williams series [33]. The question remains why SpoIIE purifies with Fe^2+^ bound rather than Mn^2+^ as these metals are next to each other and the concentration of both ions in the *E*. *coli* cytoplasm is comparable, in the range of 10^−6^ to 10^−7^ M [34]. It is not uncommon for proteins that have Mn^2+^ as cofactor to copurify with a different metal bound [34], *eg*. the *B*. *subtilis* nucleotide pyrophosphohydrolase YpgQ was recently purified and crystallized with bound Ni^2+^ although it requires Mn^2+^ for its activity [41]. It is likely that the delivery pathway for Mn^2+^ to SpoIIE requires an, as yet unknown, chaperone [34].

SpoIIE oligomerization was also induced by other divalent cations, which is not surprising as the binding specificity of enzymes for metal ions is quite low [42]. Our data indicate that domains II and III are not fully independent and may influence each other’s activity, which is in agreement with the previous *in vivo* findings, where mutations in either of the domains had an influence on the activity of the other domain [7, 15].

Manganese is important for the oligomerization and activity of SpoIIE but it has been known for a long time that it is also necessary for sporulation of *B*. *subtilis*. Mn^2+^ is an essential cofactor for phosphoglycerate phosphomutase, and in the absence of Mn^2+^ and only when cells are grown on specific carbon sources, the accumulation of 3-phosphoglycerate affects both growth and sporulation [43]. To avoid 3-phosphoglycerate accumulation we used growth media in which such accumulation does not occur, and only removed Mn^2+^ at the start of sporulation. Mn^2+^ is a cofactor for many enzymes involved in sporulation but it is not understood at which stage sporulation is blocked in the absence of Mn^2+^. Because of the role of SpoIIE in asymmetric septation, we decided to study localization of FtsZ-eYFP during sporulation in the presence and absence of Mn^2+^. We noticed that in the absence of manganese, formation of the asymmetric septum is delayed and less polar Z-rings are formed compared to cells sporulating in the presence of Mn^2+^. Only two proteins are known to be involved in the switch from medial to polar Z-ring, the sporulation-specific transcriptional factor Spo0A and SpoIIE. A mutation in the *spo0A* gene completely blocks sporulation before the formation of asymmetric septa (at stage 0). Thus, it is unlikely that the lack of manganese influences Spo0A as it would completely block relocation of the Z-ring [6]. Also, the activity of the P_spo0A_ promoter is unaffected by the absence of Mn^2+^ (S3 Fig). Localization of FtsZ in the absence of Mn^2+^ during sporulation resembles the situation in *spoIIE* null mutant cells. It has been shown previously that deletion of the *spoIIE* gene affects formation of asymmetric Z-rings but does not prevent it [9, 10]. So the delay observed in our experiment could be either because SpoIIE requires Mn^2+^ for efficient FtsZ ring positioning, or because of a secondary effect as Mn^2+^ also influences downstream activation of the sporulation phoshorelay (S3 Fig) [35–37]. We hypercharged the phosphorelay by overproducing KinA [24] and found that overall, polar septation was faster, but that there still was a delay in polar septation in the absence of Mn^2+^. Combined, our results strongly suggest that the absence of manganese influences relocation of the Z-ring in a SpoIIE-dependent manner. The observation that FtsZ polymers are stabilized, and display reduced GTPase activity, in the presence of SpoIIE is in line with our suggestion that metal-dependent SpoIIE oligomerization stabilizes FtsZ filaments in such a way that they can spiral out to form the asymmetric septum.

## Supporting information

S1 Table Percentages represented in the piechart in Fig 3. The table shows the average and standard deviation (avg ±stdv) per class scored for two independent experiments. In each single experiment at least 290 cells were scored per condition.
(PDF)

S2 Table Percentages represented in the piechart in S4 Fig. The table shows the average and standard deviation (avg ±stdv) of two independent experiments. In each single experiment at least 200 cells were scored per condition.
(PDF)

S1 Fig Absorption spectra of purified ms-SpoIIE_cyt_ (A), the same material to which 2 mM EDTA was added, after incubation for 24 hours (B), and the same material after MnCl_2_ was added to 4 mM, after incubation for 16 hrs (C). Additions resulted in slight dilution of the protein sample, from 12.1 mg/mL (A) to 11.3 mg/mL (B) to 10.3 mg/mL (C). Spectra were taken using a Nanodrop ND-1000 from 220 to 750 nm and the absorption was depicted as absorption calculated for a optical pathlength of 10 mm.
(PDF)

S2 Fig Light scattering measurement of 1,5 μM s-SpoIIE_cyt_ in the presence of 10 mM MnCl_2_ or buffer (control) added after 60 sec. (PDF)

S3 Fig The activation of the sporulation phosphorelay is delayed in the absence of Mn^2+^. Strains 168 (wt, square symbols, as reference), IDJ006 (*P*_*spo0A*_*-gfp*, triangles) and IDJ007 (*P*_*spoIIA*_*-gfp*, circles) were sporulated in the presence (closed symbols, +) and absence (open symbols, -) of Mn^2+^ and the promoter activity was monitored by following GFP fluorescence corrected for cell density. The increase of Pspo0A driven GFP production starts after roughly 60 minutes and is independent of Mn^2+^ (panel A), whereas *P*_*spoIIA*_ driven GFP production is clearly delayed in the absence of Mn^2+^ (panel B). Please note the difference in scale between panels A and B, reflective of the stronger promoter activity of *P*_*spoIIA*_. Wild type cells (same data plotted in panels A and B) were included as a reference for background fluorescence. The average and standard deviations are plotted for triplicate measurements of two biological duplicates (6 measurements each point).
(PDF)

S4 Fig The absence of Mn^2+^ from the sporulation medium delays asymmetric Z-ring formation in cells with a hypercharged phosphorelay. Pie-chart representation of sporulating *B*. *subtilis* cells in the presence (plus) and absence (minus) of Mn^2+^. Cells were scored as described for Fig 3. Non-sporulating cells are marked in blue shades, while sporulating cells are marked in orange shades. Each pie chart is the result of two independent classification experiments in which at least 200 cells were classified per experiment. Actual percentages and standard deviations are included in S2 Table.
(PDF)

S5 Fig (A) Light scattering signal of FtsZ in the presence of 2 mM GTP and 10 mM of divalent cations (Mn^2+^, Mg^2+^) or 1 mM EDTA. (B) continuation of the signal from the sample with Mn^2+^. (C, D, E) Electron microscopy of FtsZ polymers assembled in the presence of 2 mM GTP and either Mn^2+^ (C), EDTA (D) or Mg^2+^ (E) after 30 min of incubation. (F) Representative picture of FtsZ polymers assembled in the presence of 2 mM GTP and Mn^2+^ after 90 min of incubation. Scale bar 50 nm.
(PDF)
