# Supplementary material for: Metal-dependent SpoIIE oligomerization stabilizes FtsZ during asymmetric division in Bacillus subtilis
Source: PLoS One. 2017 Mar 30;12(3):e0174713. doi: 10.1371/journal.pone.0174713 (PMC5373596; doi:10.1371/journal.pone.0174713)
Supplement: S1 Table — The table shows the average and standard deviation (avg ±stdv) per class scored for two independent experiments. In each single experiment at least 290 cells were scored per condition. (PDF) [file pone.0174713.s001.pdf]

S1 Table

	No-ring		Mid-cell ring		Two polar rings		Polar ring	
	+	-	+	-	+	-	+	-
2h	29.1 ±1.8	46.8 ±1.6	5.9 ±0.6	10.0 ±3.0	43.5 ±0.8	27.0 ±2.0	21.5 ±0.3	16.2 ±0.5
3h	42.5 ±2.9	51.1 ±1.4	5.7 ±0.2	11.6 ±1.3	28.3 ±1.0	17.6 ±6.3	23.6 ±1.6	19.8 ±3.6

S1 Table. Percentages represented in the piechart in Fig. 3. The table shows the average and standard deviation (avg ±stdv) per class scored for two independent experiments. In each single experiment at least 290 cells were scored per condition.
