# Supplementary material for: Metal-dependent SpoIIE oligomerization stabilizes FtsZ during asymmetric division in Bacillus subtilis
Source: PLoS One. 2017 Mar 30;12(3):e0174713. doi: 10.1371/journal.pone.0174713 (PMC5373596; doi:10.1371/journal.pone.0174713)
Supplement: S2 Table — The table shows the average and standard deviation (avg ±stdv) of two independent experiments. In each single experiment at least 200 cells were scored per condition. (PDF) [file pone.0174713.s002.pdf]

S2 Table

	No-ring		Mid-cell ring		Two polar rings		Polar ring	
	+	-	+	-	+	-	+	-
1h	54.7 ±10.7	55.6 ± 1.5	8.6 ±0.3	15.2 ±3.4	13.7 ±0.0	14.3 ±1.5	23.0 ±10.4	15.0 ±3.4
2h	61.1 ±3.4	80.4 ±19.2	1.0 ±1.4	0.5 ±0.7	6.9 ±2.2	3.6 ±3.7	31.0 ±0.2	15.0 ±15.5
3h	84.5 ±17.7	83.7 ±10.2	0.9 ±1.2	0.2 ±0.3	4.4 ±6.2	4.3 ±2.4	10.3 ±10.3	11.8 ±7.5

S2 Table Percentages represented in the piechart in S4 Fig. The table shows the average and standard deviation (avg ±stdv) of two independent experiments. In each single experiment at least 200 cells were scored per condition.
