# Supplementary figures and images for: Metal-dependent SpoIIE oligomerization stabilizes FtsZ during asymmetric division in Bacillus subtilis
Source: PLoS One. 2017 Mar 30;12(3):e0174713. doi: 10.1371/journal.pone.0174713 (PMC5373596; doi:10.1371/journal.pone.0174713)

A

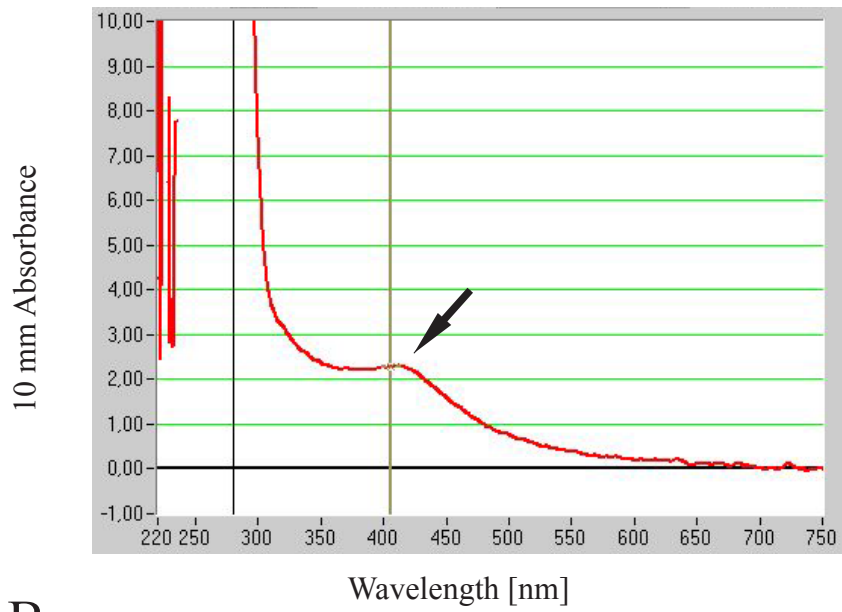

B

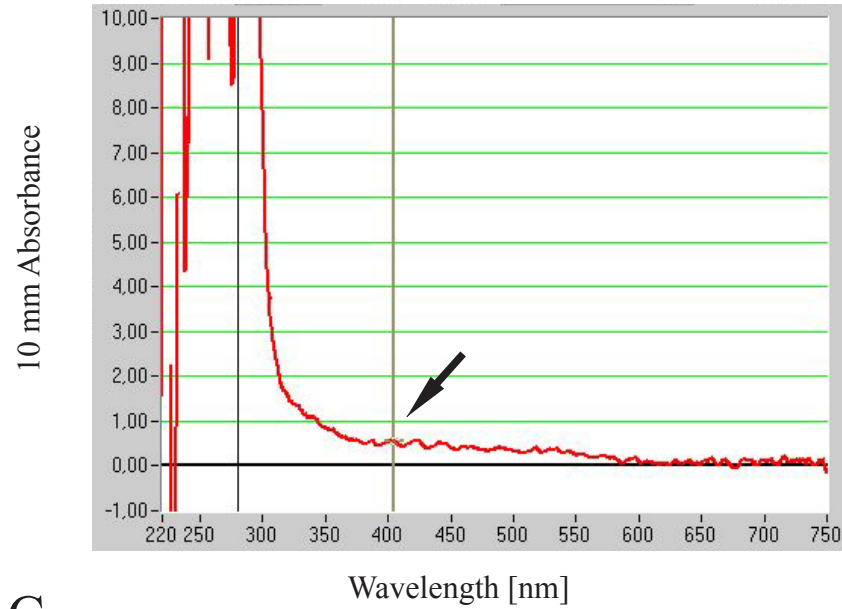

C

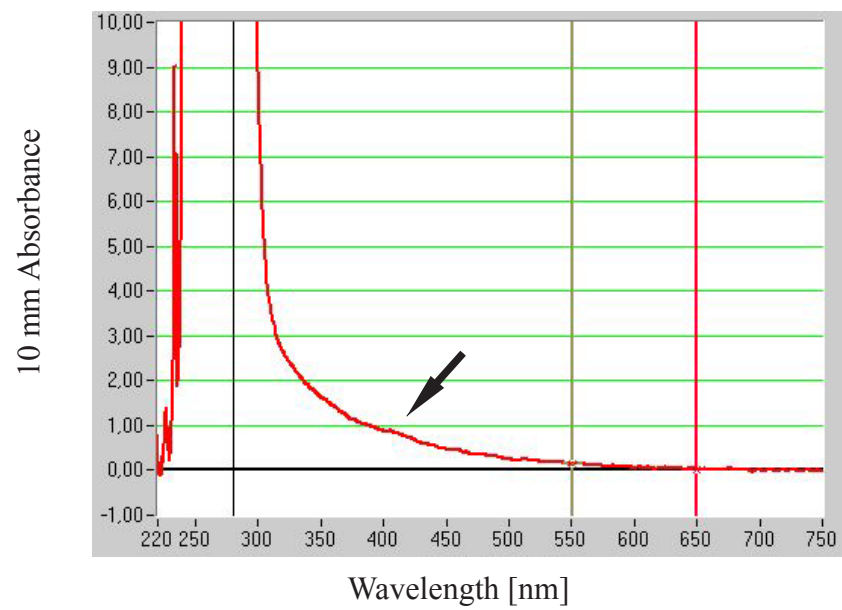

Supplement: S1 Fig — Additions resulted in slight dilution of the protein sample, from 12.1 mg/mL (A) to 11.3 mg/mL (B) to 10.3 mg/mL (C). Spectra were taken using a Nanodrop ND-1000 from 220 to 750 nm and the absorption was depicted as absorption calculated for a optical pathlength of 10 mm. (PDF) [file pone.0174713.s003.pdf]

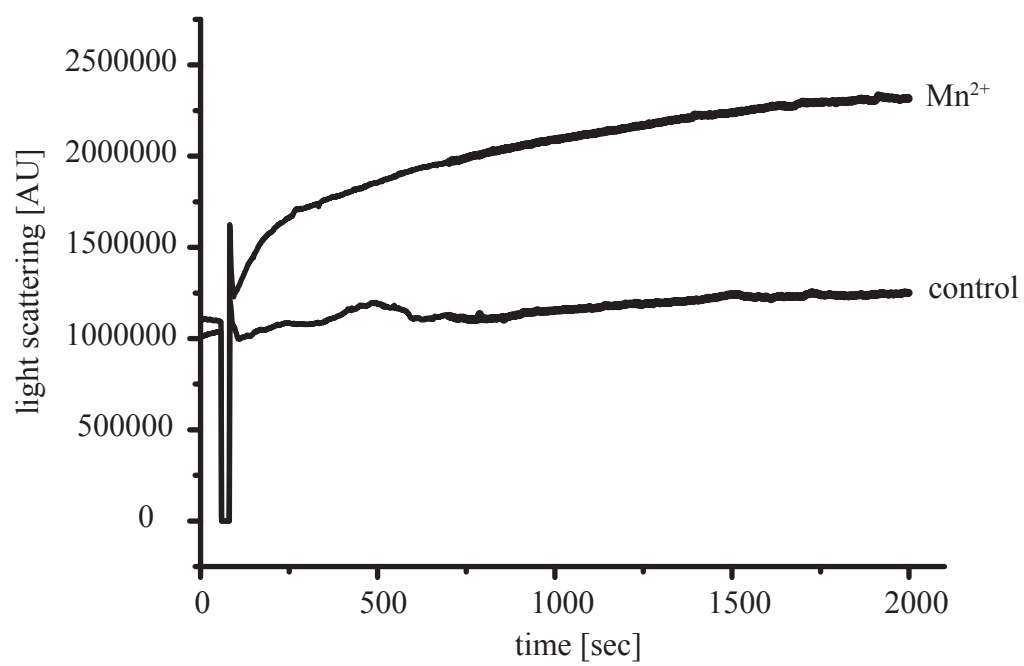

Supplement: S2 Fig — (PDF) [file pone.0174713.s004.pdf]

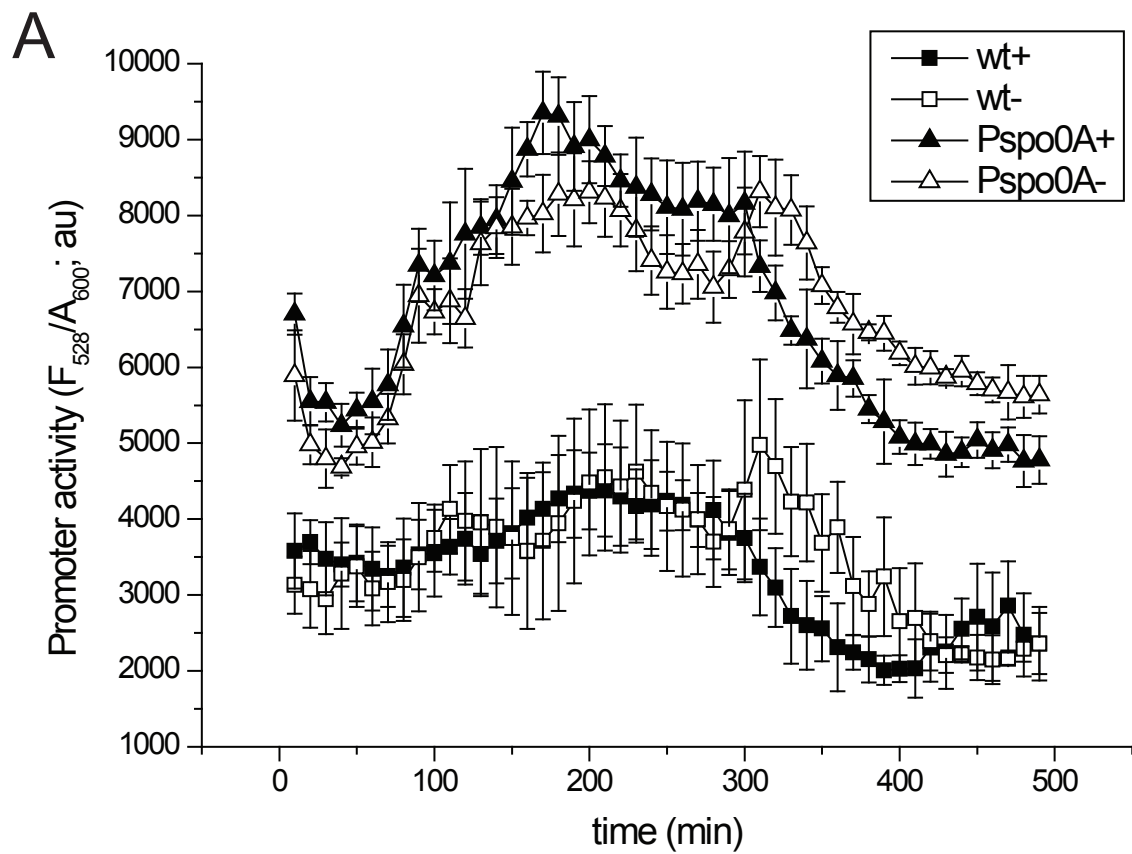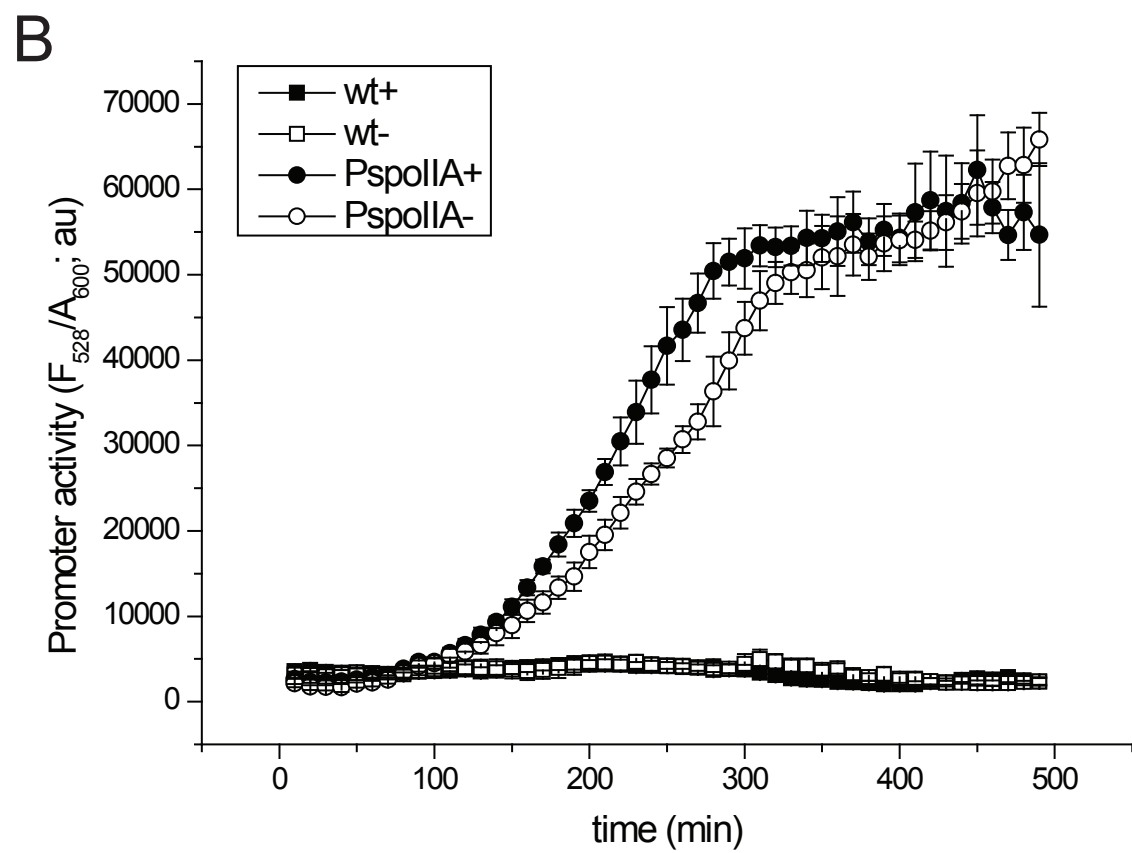

Supplement: S3 Fig — Strains 168 (wt, square symbols, as reference), IDJ006 (Pspo0A-gfp, triangles) and IDJ007 (PspoIIA-gfp, circles) were sporulated in the presence (closed symbols, +) and absence (open symbols, -) of Mn2+ and the promoter activity was monitored by following GFP fluorescence corrected for cell density. The increase of Pspo0A driven GFP production starts after roughly 60 minutes and is independent of Mn2+ (panel A), whereas PspoIIA driven GFP production is clearly delayed in the absence of Mn2+ (panel B). Please note the difference in scale between panels A and B, reflective of the stronger promoter activity of PspoIIA. Wild type cells (same data plotted in panels A and B) were included as a reference for background fluorescence. The average and standard deviations are plotted for triplicate measurements of two biological duplicates (6 measurements each point). (PDF) [file pone.0174713.s005.pdf]

plus

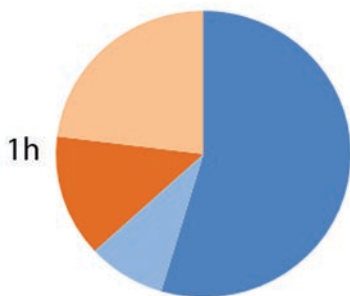

minus

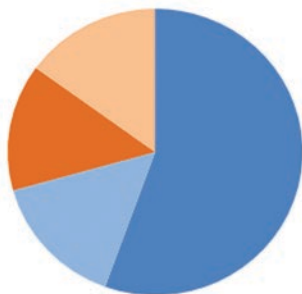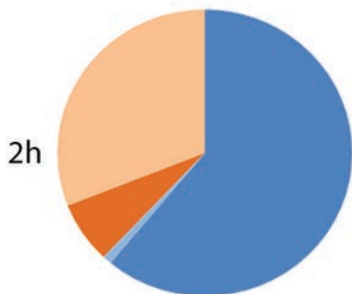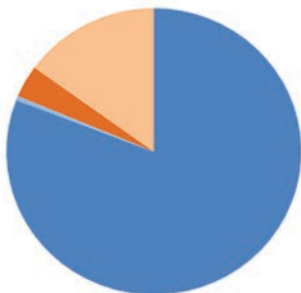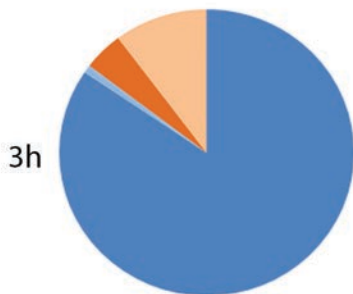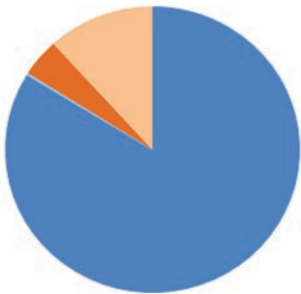

Supplement: S4 Fig — Pie-chart representation of sporulating B. subtilis cells in the presence (plus) and absence (minus) of Mn2+. Cells were scored as described for Fig 3. Non-sporulating cells are marked in blue shades, while sporulating cells are marked in orange shades. Each pie chart is the result of two independent classification experiments in which at least 200 cells were classified per experiment. Actual percentages and standard deviations are included in S2 Table. (PDF) [file pone.0174713.s006.pdf]

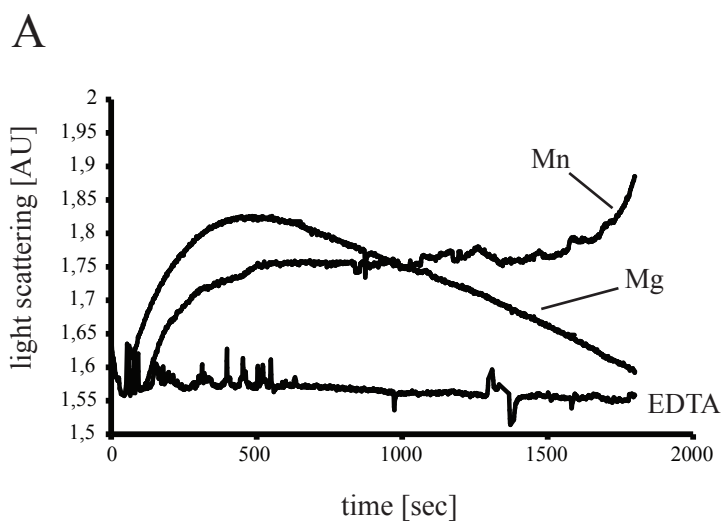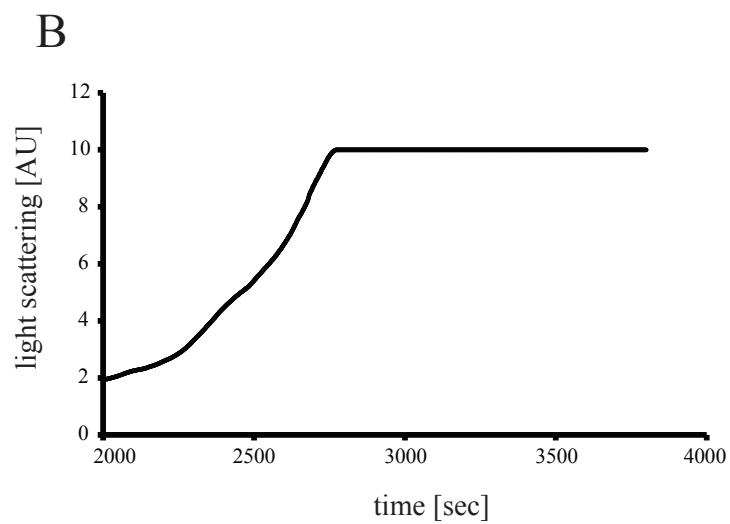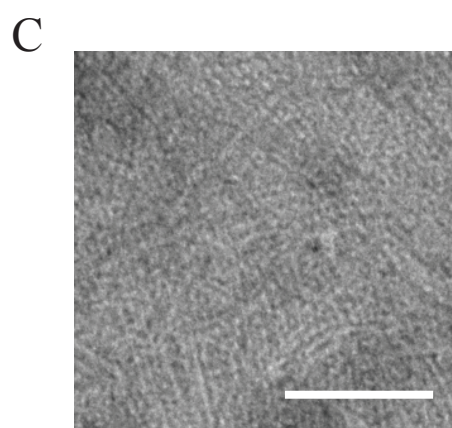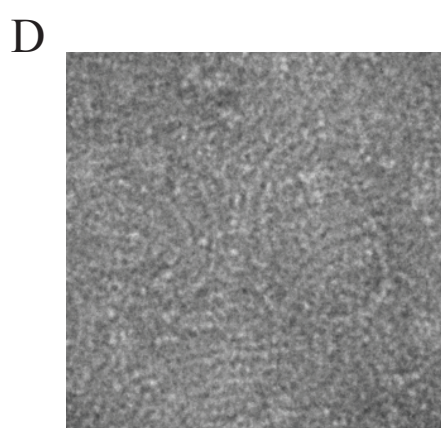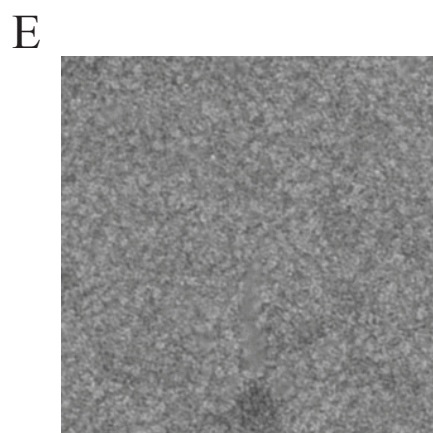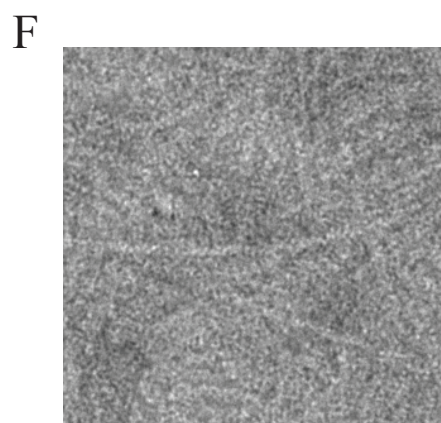

Supplement: S5 Fig — (A) Light scattering signal of FtsZ in the presence of 2 mM GTP and 10 mM of divalent cations (Mn2+, Mg2+) or 1 mM EDTA. (B) continuation of the signal from the sample with Mn2+. (C, D, E) Electron microscopy of FtsZ polymers assembled in the presence of 2 mM GTP and either Mn2+ (C), EDTA (D) or Mg2+ (E) after 30 min of incubation. (F) Representative picture of FtsZ polymers assembled in the presence of 2 mM GTP and Mn2+ after 90 min of incubation. Scale bar 50 nm. (PDF) [file pone.0174713.s007.pdf]
